# Detection of human bocavirus from children and adults with acute respiratory tract illness in Guangzhou, southern China

**DOI:** 10.1186/1471-2334-11-345

**Published:** 2011-12-14

**Authors:** Wen-Kuan Liu, De-Hui Chen, Qian Liu, Huan-Xi Liang, Zi-Feng Yang, Sheng Qin, Rong Zhou

**Affiliations:** 1 State Key Laboratory of Respiratory Diseases, Guangzhou Medical University, 1 Kang Da Road, Guangzhou, Guangdong 510230, China; 2 The First Affiliated Hospital of Guangzhou Medical University, 151 Yan Jiang Road, Guangzhou, Guangdong 510230, China

## Abstract

**Background:**

Human bocavirus (HBoV) is a newly discovered parvovirus associated with acute respiratory tract illness (ARTI) and gastrointestinal illness. Our study is the first to analyze the characteristics of HBoV-positive samples from ARTI patients with a wide age distribution from Guangzhou, southern China.

**Methods:**

Throat swabs (n=2811) were collected and analyzed from children and adults with ARTI over a 13-month period. The HBoV complete genome from a 60 year-old female patient isolate was also determined.

**Results:**

HBoV DNA was detected in 65/2811 (2.3%) samples, of which 61/1797 were from children (<18 years old) and 4/1014 from adults (≥18 years old). Seasonal peaks of 4.8% and 7.7% were detected in May and June, respectively. 28 of 65 (43.1%) HBoV-positive samples were co-detected with 11/16 other potential pathogens. *Mycoplasma pneumoniae *had the highest frequency of 16.9% (11/65). Upper and lower respiratory tract illness were common symptoms, with 19/65 (29.2%) patients diagnosed with pneumonia by chest radiography. All four adult patients had systemic influenza-like symptoms. Phylogenetic analysis of the complete genome revealed a close relationship with other HBoVs, and a more distant relationship with HBoV2 and HBoV3.

**Conclusions:**

HBoV was detected from children and adults with ARTI from Guangzhou, southern China. Elderly people were also susceptive to HBoV. A single lineage of HBoV was detected among a wide age distribution of patients with ARTI.

## Background

Respiratory tract infection etiology is complex and diverse, and new pathogens are continuously being reported. Over the past few years, several novel respiratory viruses including human metapneumovirus (hMPV) [1], severe acute respiratory syndrome (SARS) coronavirus [2], human coronavirus NL63 (HCoV-NL63) [3,4], and coronavirus HKU1 (HCoV-HKU1) [5-7] have been identified.

In 2005, Allander et al. [8] reported a previously undescribed human parvovirus, human bocavirus (HBoV) that belongs to the genus *Bocavirus*, in respiratory secretions of children with respiratory tract disease in Sweden. HBoV is a single-stranded deoxyribonucleic acid (DNA) virus with a small genome size of approximately 5.3 kilo-bases (kb), which has three open reading frames (ORF) encoding two non-structural proteins NS1 and NP1, and the two structural proteins VP1 and VP2. VP1 and VP2 are located within the same ORF but have different initiator codon positions [8].

Subsequently, HBoV was reported in respiratory samples from different countries and regions worldwide [9-14], where HBoV was detected in 1.5%-8.3% of respiratory samples from individuals with acute respiratory tract illness (ARTI), especially young children and infants. The virus was also found in stool samples from patients with gastrointestinal illness [15-22]. These reports suggest that HBoV might be associated with upper and lower respiratory disease and gastrointestinal illness throughout the world. In 2009, two viruses closely related to HBoV, named HBoV2 [23] and HBoV3 [24], were found in stool samples, and suggested HBoV diversity.

HBoV infection has recently attracted increasing attention all over the world. However, the incidence and clinical presentation of this infection varies widely, and often involves co-infection with other potential pathogens [9-22]. Such characteristics have led to debate over the role of HBoV as a true pathogen. Therefore, additional evidence and studies are needed throughout the world to gain a better understanding of this virus. In this study, 2811 respiratory samples were collected from patients (with an age range of 9 days to 84 years) with ARTI in Guangzhou, southern China, from November 2009 to November 2010 to analyze the characteristics of HBoV-positive patients.

## Methods

Samples in this study were taken as part of standard care. The First Affiliated Hospital of Guangzhou Medical University Ethics Committee approved the experimental design and patient involvement in this study.

### Respiratory samples collection

Throat swab samples (n = 2811) were collected from patients with ARTI (presented at least two of the following symptoms: cough, pharyngeal discomfort, rhinobyon, snivel, sneeze, dyspnea) at three hospitals in Guangzhou, southern China between November 2009 and November 2010. Patients' ages ranged from nine days to 84 years, and included 1797 children (<18-years-old) and 1014 adults (≥18-years-old). Clinical characteristics of the patients were recorded for further analysis.

### Real-time polymerase chain reaction (PCR) for HBoV detection

DNA from respiratory samples was extracted using a QIAamp DNA Mini Kit (Qiagen), in accordance with the manufacturer's protocol. Taqman real-time PCR primers and probe were designed based on the conserved region of the NP1 gene. Sequences were as follows: forward primer, 5'- GAG AGA GGC TCG GGC TCA TA-3' (2545-2564 nt); reverse primer, 5'- TCG AAG CAG TGC AAG ACG AT-3' (2592-2611 nt); and probe, 5'-FAM- CAT CAG GAA CAC CCA ATC AGC CAC C-BHQ1-3' (2566-2590 nt). Primers and the probe were synthesized by TaKaRa. Premix Ex Taq (Perfect Real Time) real-time PCR reaction buffer was also purchased from TaKaRa. Amplification was conducted using 10 pmol of primers, 3 pmol of probe and 5 μl DNA in a final volume of 25 μl. Cycling conditions included an initial incubation at 94°C for 2 min, followed by 40 cycles of 94°C for 10 sec and 55°C for 35 sec (ABI-7500 real-time PCR instrument,Applied Biosystems). The amplified NP1 gene target sequence (2545-2611 nt) was inserted into the pMD18-T vector (TaKaRa) and used as a positive control for quantification analysis. Sensitivity of the PCR assay was calculated to be 10 copies of plasmid DNA using positive control plasmid diluted gradients.

### Detection of common respiratory pathogens in HBoV-positive samples

HBoV DNA positive samples were tested for 16 other potential pathogens, including influenza A virus, influenza B virus, parainfluenza virus (1, 2, 3, 4), respiratory syncytial virus, adenovirus, enterovirus, human metapneumovirus, human coronavirus (229E, OC43, NL63, HKU1), *Mycoplasma pneumoniae*, and *Chlamydia pneumoniae *by Taqman real-time PCR, in accordance with the manufacturer's protocol (Guangzhou HuYanSuo Medical Technology Co., Ltd).

### Complete genome sequencing

The complete genome of HBoV from a 60-year-old female patient isolate was sequenced and analyzed. Sequencing primer sets were designed according to HBoV genome sequences available in the GenBank database (Table 1). DNA template (2 μl) was added to the Pfu DNA polymerase reaction mixture (Fermentas) in a final volume of 25 μl and PCR amplified. Products were purified after 1% agarose gel electrophoresis using a QIAquick Gel Extraction Kit (Qiagen). The purified DNA was then sequenced (ABI Genetic Analyzer 3130XL) and assembled using DNASTAR-SeqMan software (DNASTAR, http://www.dnastar.com/t-products-lasergene.aspx). PCR amplification and sequencing were conducted at least twice to ensure sequence accuracy.

**Table 1:** Primer sets for HBoV genome sequencing

Primers	Sequences	Amplification position (bases)	Product length
VF1	5'-GCCGGCAGACATATTGGA-3'	1-1460	1460bp
		
VR1	5'-TGACCAACGGCTAGAGGATTA-3'		
		
VF2	5'-ATGCTAAATCATCCTGTG-3'	638-2435	1798bp
		
VR2	5'-TTGTCTTTCATATTCCCT-3'		
		
VF3	5'-ACCCAAGAAACGTCGTCTAACTG-3'	2300-4226	1927bp
		
VR3	5'-TTAGTCCAGGAGGAATGTATGCT-3'		
		
VF4	5'-GGAGGCAATGCTACAGAAA-3'	4064-5299	1236bp
		
VR4	5'-TGTACAACAACAACACATTAAAAG-3'		

### Phylogenetic analysis

The HBoV complete genome (GU338055) was aligned to other HBoV genomes available in the GenBank database using the Basic Local Alignment Search Tool (BLAST, http://blast.ncbi.nlm.nih.gov/Blast.cgi). Phylogenetic analysis of 18 complete genomes of HBoV, HBoV2 and HBoV3 from different countries and regions, including USA, Sweden, Thailand, Japan, Australia, China, Hong Kong, and Taiwan was conducted using Molecular Evolutionary Genetics Analysis Version 4.0 (MEGA 4.0, http://www.megasoftware.net/). Phylogenetic trees were inferred from VP1/VP2 (3056-5071 nt), NS1 (253-2172 nt), NP1 gene (2410-3069 nt) and complete genome data (1-5299 nt) using the neighbor-joining method, and bootstrap values were calculated from 1000 replicates.

### Statistical analysis

For comparison of categorical data, χ^2 ^test and Fisher's exact test where appropriate. All tests were two-tailed and *p *< 0.05 was considered statistical significant.

## Results

### Detection of HBoV from patients with ARTI

**Figure 1:**
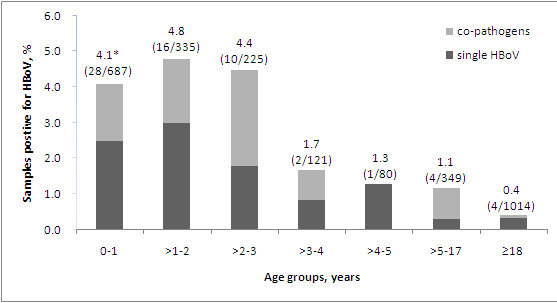
**Distribution of HBoV positive patients among different age groups**. *Positive rate (%). The number of HBoV-positive/total patients in each age groups are listed in parenthesis. Co-pathogens: co-detected with other pathogens. Single HBoV: detected with single HBoV, without other 16 potential pathogens.

HBoV DNA positive samples were detected in 65/2811 patients with a total positive rate of 2.3%. In children, positive rates were 4.1% (28/687) for 0-1 year-olds, > 4.8% (16/335) for 1-2 year-olds, 4.4% (10/225) for >2-3 year-olds, 1.7% (2/121) for >3-4 year-olds, 1.3% (1/80) for >4-5 year-olds, and 1.1% (4/349) for >5-17 year-olds (Figure 1), with a total of 3.4% (61/1797). The positive rate of adults (≥18 year-old) was 0.4% (4/1014) (Figure 1).

The 65 positive patients were aged between 54 days and 70 years, comprising 83.1% (54/65) that were ≤ 3 years-old, 10.8% (7/65) that were 4-17 years-old, and 6.2% (4/65) adult patients. Four HBoV-positive adults were 19, 22, 60 and 70 years-old, respectively.

### Seasonal distribution of HBoV

**Figure 2:**
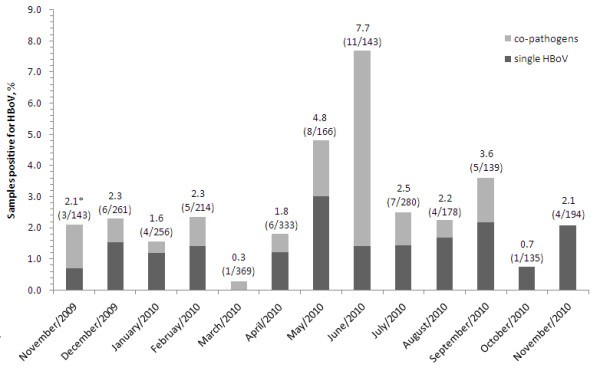
**Seasonal distribution of HBoV from November 2009 to November 2010**. *Positive rate (%). The number of HBoV-positive/total patients in 13 months are listed in parenthesis. Co-pathogens: co-detected with other pathogens. Single HBoV: detected with single HBoV, without other 16 potential pathogens.

During our 13-month study period, more than 100 samples were collected for detection each month, and the ratio of ≤3 year-old patients ranged from 20.2% to 66.5%. Low positive rates of 0.3% (1/369) (single HBoV: 0, co-pathogen: 0.3%) were detected in March 2010 and 0.7% (1/135) (single HBoV: 0.7%, co-pathogen: 0) in October 2010, while high positive rates of 4.8% (8/166) (single HBoV: 3.0%, co-pathogens: 1.8%) were found in May 2010 and 7.7% (11/143) (single HBoV: 1.4%, co-pathogens: 6.3%) in June 2010 (Figure 2). The positive rates of other seven months fluctuated around the total rate of 2.3% (65/2811) (Figure 2).

### Co-detection with common respiratory pathogens

HBoV DNA positive samples were co-detected with 11 of 16 upper referred pathogens in 28/65 (43.1%) of the patients. *Mycoplasma pneumoniae *had the highest frequency of 16.9% (11/65), followed by RSV with 7.7% (5/65) (Table 2).

**Table 2:** Co-pathogens of HBoV-positive patients

Co-pathogens	Patient No. (%)
*Mycoplasma pneumoniae*	11 (16.9)

Respiratory syncytial virus	5 (7.7)

Parainfluenza virus type 3	4 (6.2)

Enterovirus	4 (6.2)

Influenza A virus	3 (4.6)

Human coronavirus OC43	3 (4.6)

Human coronavirus HKU1	3 (4.6)

Adenovirus	2 (3.1)

Human metapneumovirus	2 (3.1)

Influenza B virus	1 (1.5)

Parainfluenza virus type 1	1 (1.5)

### The clinical characteristics of HBoV-positive patients

The male:female ratio was 42:23 in the HBoV-positive patients which did not differ significantly from the HBoV-negative patients (*p *= 0.40). The clinical characteristics of the patients are listed in Table 3. Most patients presented with symptoms of upper respiratory tract illness (URTI), including cough (93.8%) and expectoration (40.0%); 44 (67.7%) patients presented with symptoms of fever (≥38°C); 41 (63.1%) patients had lower respiratory tract illness symptoms (LRTI) and 19 (29.2%) patients were diagnosed as pneumonia by chest radiography; 10 (15.4%) patients had gastrointestinal symptoms; seven (10.8%) patients had systemic influenza-like symptoms (chilly, swirl, headache, myodynia or debilitation); and one (1.5%) patient had herpetic angina.

**Table 3:** Clinical characteristics of HBoV-positive patients

Characteristics	Single HBoV^a^	Co-pathogens	Total	p values^b^
Patients No.	37	28	65	

Cough	34 (91.9)	27 (96.4)	61 (93.8)	0.451

Fever (≥38°C)	24 (64.9)	20 (71.4)	44 (67.7)	0.575

Abnormal pulmonary breathing sound ^c^	23 (62.2)	12 (42.9)	35 (53.8)	0.122

Dyspnea	19 (51.4)	11 (39.3)	30 (46.2)	0.334

Expectoration	17 (45.9)	9 (32.1)	26 (40.0)	0.261

Snivel	13 (35.1)	10 (35.7)	23 (35.4)	0.961

Pneumonia	6 (16.2)	13 (46.4)	19 (29.2)	**0.008**

Rhinobyon	5 (13.5)	9 (32.1)	14 (21.5)	0.070

Vomiting	3 (8.1)	3 (10.7)	6 (9.2)	0.719

Sneeze	3 (8.1)	2 (7.1)	5 (7.7)	0.885

Pharyngeal discomfort ^d^	3 (8.1)	2 (7.1)	5 (7.7)	0.885

Poor appetite	3 (8.1)	1 (3.6)	4 (6.2)	0.451

Chill	3 (8.1)	0 (0)	3 (4.6)	0.123

Diarrhea	1 (2.7)	2 (7.1)	3 (4.6)	0.398

Lung markings increasing	1 (2.7)	1 (3.6)	2 (3.1)	0.841

Headache	1 (2.7)	1 (3.6)	2 (3.1)	0.841

Myodynia	2 (5.4)	0 (0)	2 (3.1)	0.211

Debilitation	1 (2.7)	1 (3.6)	2 (3.1)	0.841

Herpangina	1 (2.7)	0 (0)	1 (1.5)	0.381

Swirl	1 (2.7)	0 (0)	1 (1.5)	0.381

^a^Data are No. (%) of each group, percentages sum to >100% because some patients had >1 diagnosis.

^b^Statistical difference between "Single HBoV" and "Co-pathogens"

^c^Including phlegm rale, wheeze rale, bubbling rale, moist rale, and rhonchi.

^d^Including pharyngeal dryness, pharyngalgia, and trachyphonia.

Co-pathogens: co-detected with other 16 potential pathogens.

Single HBoV: detected with single HBoV, without other 16 potential pathogens.

Abnormal pulmonary breathing sounds and dyspnea were detected in 35 (53.9%) and 30 (46.2%) of 65 patients, respectively (Table 3). Nineteen (29.2%) were diagnosed as pneumonia by chest radiography, and 13/19 (68.4%) pneumonia patients were co-detected with one or two other pathogens, and a statistic difference was found for the symptom of "pneumonia" (*p *= 0.008) between the two groups "Single HBoV" and "Co-pathogens". Two of seven patients with systemic influenza-like symptom samples were co-detected with influenza A virus-enterovirus (triple pathogens) and influenza B virus (dual pathogens), respectively, while the remaining five patients were detected with single HBoV. All four adult patients (≥18 years old) presented with systemic influenza-like symptoms; three had only HBoV detection and the other 19 year-old female patient was co-detected with influenza B virus.

### Complete genome sequencing and phylogenetic analysis

**Figure 3:**
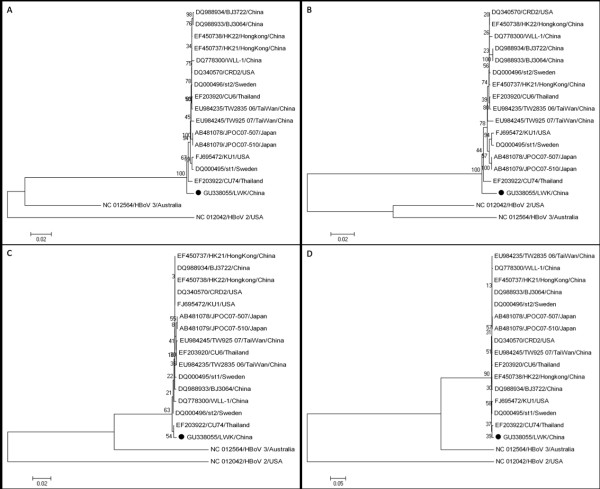
**Phylogenetic analysis of HBoV-GU338055 genome (A), VP1/VP2 genes (B), NP1 gene (C) and NS1 gene (D). HBoV/GU338055 is indicated with "●"**.

Complete HBoV genome sequences for isolates from a 60 year-old patient were sequenced and submitted to the GeneBank (Accession Number:GU338055). The full length of the genome was 5299 bases and the distribution of A/G/C/T was 32.4%/20.4%/21.8%/25.4%, respectively. Compared to the other HBoV genomes available in the GeneBank database, GU330588 showed a 98% similarity with the HBoV previously described by Allander et al. [8]. Phylogenetic trees were inferred from VP1/VP2, NS1, and NP1 gene data, in addition to the complete genome sequence (Figure 3). GU330588 was similar to other HBoVs, although it displayed obviously sequence variations from HBoV2 and HBoV3. Three HBoV lineages were illustrated in all four phylogenetic trees (Figure 3).

## Discussion

HBoV is a novel parvovirus first described in 2005 by Allander and colleagues [8]. Since that time, it has been associated with upper and lower-respiratory tract disease and gastrointestinal illness throughout the world. However, most studies were focused on young children and infants [9-14], and only a few papers have described the characteristics of HBoV infection in adult patients [16,25,26]. Our study successfully analyzed the characteristics of HBoV-positive samples from ARTI-infected patients with a wide age distribution from Guangzhou, southern China for the first reported time. Similar to previous study [16], the detection rate in pediatric patients (<18 years old) was significantly higher than that in adults (≥18 years old) (*p *< 0.001), and most HBoV-positive patients were ≤3 years old. HBoV was detected in four adult patients, including 60 and 70 year-old patients. This suggested that older people were also susceptive to HBoV infection, although at much lower positive rates. Four adult patients also presented with systemic influenza-like symptoms, which might suggest that HBoV infection in adults is a more complex and serious problem than in children.

While seasonal peaks of HBoV infection vary among different counties and regions because of climate and other factors, many previous studies suggest a higher detection rate in winter [27,28]. In our study, a higher frequency of HBoV was observed between May and June during the 13-month testing period (Figure 2). This result was similar to the report of Choi and colleagues, in which a seasonal peak was observed between May and June [29].

Characteristics of HBoV-positive patients in our study were also similar to previous reports [9-14]. The male:female ratio in the HBoV-positive patients did not differ significantly from the HBoV-negative patients. In all clinical characteristics, seven major symptoms (cough, fever (≥38°C), abnormal pulmonary breath sound, dyspnea, expectoration, snivel and pneumonia) had the highest ratio (ratio > 10%) (Table 3) and were common as URTI and LRTI.

As previous studies [13,29-34], co-detection with other potential pathogens was common in HBoV-positive patients. In this work, 43.1% (28/65) patients were co-detected with other 16 potential pathogens, and there would be higher ratio if human rhinovirus were concerned [16]. Furthermore, not only co-pathogens but also single HBoV groups had a high ratio of major symptoms (Table 3), which might suggest HBoV is an important pathogen in URTI and LRTI. A statistic difference was found for the symptom of "pneumonia" between the two groups "Single HBoV" and "Co-pathogens" (Table 3), which might further suggest HBoV is an important pathogen. Further studies were required to determine whether HBoV played a causative role in these co-infections or acted as an exacerbation factor.

Before 2009, little variation was found in the surface protein of HBoV (VP1-VP2), and some researchers predicted that HBoV infection might only occur after the subsequently development of life-long immunity via the neutralization of the target antibody [16]. However, novel types of HBoV2 and HBoV3 were described successively in 2009 [23,24], suggested diversity within this group of viruses. Furthermore, we successfully sequenced the complete HBoV genome (GU338055) for an isolate from an adult patient and phylogenetic analysis revealed the existence of three lineages: group I, HBoV; group II, HBoV2; group III, HBoV3, and GU338055 was located in group I.

The frequency and clinical presentations of HBoV infection vary widely [9-14,16,25,26] and are typically associated with other pathogens [13,29-34]. Such characteristics have subsequently led many scientists to question the role of HBoV as a potential pathogen. This lack of information is largely because of the inability of HBoV to grow in standard cell lines [35]. To confirm the effects of HBoV, more studies are required throughout the world, focusing on various aspects of this infection, including epidemiology, serology, molecular biology, *in vitro *culture and animal models.

## Conclusions

HBoV was detected from children and adults with ARTI from Guangzhou, southern China, and the features were described in this study. HBoV was confirmed in elderly patients (60 and 70 years old), suggesting that older people were also susceptive to HBoV. All four adult patients with HBoV positive in this study presented systemic influenza-like symptoms, which potentially suggest that HBoV infection in adults may develop more serious symptoms than those in children. Phylogenetic analysis suggested that HBoV-GU338055 from an elderly patient is in a single lineage with other HBoVs.

## Competing interests

The authors declare that they have no competing interests.

## Authors' contributions

RZ and W-KL designed the study. W-KL, Q L, H-XL performed the HBoV DNA testing. D-HC, Z-FY and SQ collected clinical data. All authors participated in the data analysis. W-K L and R Z drafted the manuscript. All authors read and approved the final version of this manuscript.

## Pre-publication history

The pre-publication history for this paper can be accessed here:

http://www.biomedcentral.com/1471-2334/11/345/prepub
